# The Rural Ecological Framework of Intimate Partner Violence (RE‐IPV): Social–Ecological Factors and Constrained Agency Among Rural Women

**DOI:** 10.1111/nin.70184

**Published:** 2026-11-01

**Authors:** Frances Kimber, Hanan Abusbaitan, Leso Munala, Jeneile Luebke, Angelica Walton, Anwar Eyedat, Kaboni W. Gondwe, Katie Bement, Missy Jerome, Cassandra Clark, Lucy Mkandawire‐Valhmu

**Affiliations:** ^1^ School of Nursing University of Minnesota Minneapolis Minnesota USA; ^2^ School of Nursing Oakland University Rochester Michigan USA; ^3^ School of Nursing University of Wisconsin Madison Wisconsin USA; ^4^ Department of Community and Mental Health, Faculty of Nursing The Hashemite University Zarqa Jordan; ^5^ School of Nursing University of Washington Seattle Washington USA; ^6^ Embrace Ladysmith Wisconsin USA

**Keywords:** constrained agency, framework, intimate partner violence, rural health, Social–Ecological Model, women's health

## Abstract

Intimate partner violence (IPV) is a pervasive public health crisis, with rural social and structural conditions creating additional challenges for survivors seeking safety and support. Existing applications of the Social–Ecological Model (SEM) have helped identify factors influencing IPV, but offer less explanation about how these factors interact to constrain survivors' agency and shape service navigation. This paper introduces the Rural Ecological Framework of Intimate Partner Violence (RE‐IPV), a conceptual framework informed by the SEM, structural violence perspectives, and a synthesis of empirical literature, with refinement through engagement with rural community partners, nurse scholars, and public health scientists. RE‐IPV illustrates how interacting societal, community, relational, and individual conditions shape the options available for rural women experiencing IPV. Central to the framework is constrained agency, which helps explain how survivors make decisions about safety, disclosure, and service utilization within circumstances that limit their available options. The framework also conceptualizes service navigation as a recursive process in which healthcare and advocacy encounters can influence survivors' trust, perceptions of safety, and subsequent help‐seeking decisions. By moving beyond a descriptive categorization of ecological barriers, RE‐IPV offers a rural‐specific perspective on how structural conditions shape survivors' agency and engagement with services. The framework provides a foundation for future conceptual development, empirical examination, and survivor‐centered nursing and healthcare practice.

**Trial Registration:** Not applicable.

## 1 Introduction: Why Rural Intimate Partner Violence Needs Ecological Rethinking

In the United States, nearly half of women experience some form of intimate partner violence (IPV) in their lifetime, making it one of the most pervasive, yet underrecognized, threats to women's health (National Academies of Sciences, Engineering, and Medicine 2024). IPV is defined as physical or sexual violence, psychological aggression (including coercion), or stalking, perpetrated by a current or former intimate partner (Breiding et al. 2015). The vast majority of women, 71%, experience their first victimization prior to 25 years of age (S. G. Smith et al. 2018), with lethal IPV accounting for nearly half of female homicides and serving as a leading driver of maternal mortality in the United States (Petrosky et al. 2017; Wallace et al. 2021).

Beyond these immediate dangers, IPV exerts profound health impacts that directly compromise a survivor's physical, cognitive, and emotional capacity to navigate help‐seeking. Head injury and traumatic brain injury (TBI), both consequences of physical abuse, may impair cognitive functioning and memory, subsequently creating additional barriers to decision‐making and help‐seeking (Smirl et al. 2019; Valera and Kucyi 2017). Other long‐term effects include chronic pain, cardiovascular and gastrointestinal disorders, mental health conditions, and reproductive complications, such as preterm birth and miscarriage (Banjar et al. 2022; Barez et al. 2025; Lagdon et al. 2014; Thurston et al. 2022; Uvelli et al. 2024). These cumulative health effects may ultimately restrict a survivor's functional bandwidth for navigating violence and paths to safety.

For women in rural America, this crisis is profoundly complicated, and often further obscured, by the unique societal, community, and relational factors present in rural settings. Evidence regarding IPV incidence in rural versus urban settings is mixed; however, research suggests that IPV in rural communities may be more severe, with higher rates of intimate partner homicide and substantial barriers to accessing support services (Edwards 2015; Peek‐Asa et al. 2011; Strand and Storey 2019). Approximately 20% of the US population resides in rural areas, accounting for 87.7% of the nation's land area. Rural communities are characterized by lower population density and geographic dispersion, and are often underserved with fewer healthcare, social service, and other community resources (Health Resources and Services Administration 2024). The challenge of attempting to remain safe and/or seek help is uniquely compounded as rural survivors face multi‐level ecologic factors specific to rural contexts. Current IPV frameworks are not rural‐centered and fail to adequately capture these unique factors and how survivors navigate this environment. To address this gap, we sought to develop a rural violence framework, guided primarily by the Social–Ecological Model (SEM), to better understand how rurality impacts survivor decision‐making and resource navigation. Through our proposed framework, we introduce three conceptual shifts: (1) the greater rural ecology shapes IPV experiences, (2) barriers within the ecology manufacture a state of constrained agency for the survivor, and (3) service navigation is a recursive process.

## 2 Existing Ecological Approaches to IPV: Contributions and Limits

### 2.1 Social–Ecological Model

The SEM was developed by psychologist Urie Bronfenbrenner as a foundational framework for understanding human development. Bronfenbrenner posited that to understand human growth, one must understand the broader ecology of that individual, which consisted of five interconnected paradigms: microsystem, mesosystem, exosystem, macrosystem, and the chronosystem (Bronfenbrenner 1993). The SEM was later adapted to gender‐based violence research to illustrate that abuse is not merely the product of isolated relationship and individual dynamics; rather, abuse originates from and is informed by various personal, situational, and sociocultural factors (Heise 1998). In contemporary IPV literature, the SEM has traditionally been utilized to categorize the unique risk and protective factors across individual, relational, community, and societal ecologies (Tekkas Kerman and Betrus 2020). By mapping variables across these separate layers, the SEM has historically allowed public health scientists to design multi‐level and targeted interventions for primary, secondary, and tertiary prevention.

Across decades of application, the standard SEM has, however, remained largely static and descriptive. It excels at cataloging what factors correlate with and perpetuate violence, but it fundamentally lacks a mechanical explanation of how these layers actively interact to restrict a survivor's autonomy and the subsequent outcomes. Traditional applications of the SEM in IPV literature have predominantly focused on social and interpersonal ecologies, frequently failing to account for the physical and environmental ecosystems such as resource scarcity, geographic isolation, and infrastructure deficits that actively strain survival (Abusbaitan et al. 2026). Other applications of SEM in IPV research are often generalized without a rural‐specific focus (Ogolsky et al. 2026; Shorey et al. 2023). There remains a critical gap in our theoretical understanding of how a survivor's help‐seeking is influenced and constrained by their unique rural ecology in the face of violence.

### 2.2 Alternative IPV Frameworks

Beyond the classical SEM, critical feminist paradigms, including intersectional, decolonial, and patriarchy‐focused frameworks, have offered essential insights into structural drivers of violence. Early feminist theorizing established that IPV stems from systemic patriarchal power dynamics and state‐sanctioned gender inequality rather than individual pathology (Dobash 1979). Intersectional feminist theory expanded this lens to illustrate how gendered oppression interacts simultaneously with race, class, and marginalized social positions (Crenshaw 1989). Similarly, decolonial feminist frameworks demonstrate how ongoing colonial structures and historical trauma systemically degrade collective protection systems and institutional reliability (Luebke et al. 2021; L. T. Smith 2012); however, while these perspectives offer macro‐level critiques, they frequently operate from an implicit urban bias, overlooking the distinct spatial and material realities of rural survival. Moreover, help‐seeking frameworks in the IPV literature often over‐emphasize cognitive or individual decision‐making stages without fully operationalizing how rural structural barriers and environmental precarity actively restrict the availability of viable options.

## 3 Framework Development

### 3.1 Conceptual Development and Refinement

The proposed framework, titled Rural Ecological Framework of Intimate Partner Violence (RE‐IPV), was developed through an interactive synthesis of theoretical and empirical scholarship examining societal, community, relational, and individual influences on help‐seeking and access to support services. The synthesis drew on scholarship addressing IPV, rural women's health, constrained agency, service navigation, and social determinants of health, with particular attention to rural ecological conditions including cultural norms, geographic isolation, healthcare accessibility, transportation, poverty, gender norms, law enforcement responses, confidentiality concerns, and social support. Foundational scholarship on the SEM and structural violence provided additional conceptual grounding. The emerging framework was refined through collaborative discussion within the research team, including consideration of the organization of constructs and their interrelationships.

Concepts identified through this synthesis were considered in relation to the corresponding levels of the SEM, with particular attention to relationships across ecological levels and their implications for survivors' help‐seeking and service navigation.

The initial model underwent further refinement through review by an interdisciplinary panel of eight experts, including nurse scholars, to validate individual and health system factors; public health scientists to validate the structural infrastructure and geographic constraint components; and violence content experts to assess the dynamics of coercive control and interpersonal elements of IPV. Importantly, three rural community advocates reviewed our findings and model at multiple points in development to ensure the framework accurately reflected the lived reality of survivors and the barriers they face. Feedback from these experts guided modifications, ultimately creating the reconfigured, rural‐specific framework. The framework was also informed by insights gained from our US‐based National Institutes of Health (NIH) R01 study (R01MD016388). This large‐scale mixed‐methods study examined help‐seeking and safety among 282 women in the Upper Midwest who experienced IPV during the COVID‐19 pandemic. Experiences among rural participants, including geographic isolation, limited service availability, and restrictive community norms and stigma, contributed to the conceptualization of rural ecological constraints represented in RE‐IPV.

### 3.2 Author Positionality

The authors are a multidisciplinary and culturally diverse team of nursing scholars, public health scientists, and IPV advocates. The research team comprises Black, White, Arab, and American Indian scholars and advocates whose professional and lived perspectives of working with IPV survivors inform a deeper understanding of IPV in rural settings. Throughout the conceptual development of RE‐IPV, the authors considered how their disciplinary backgrounds, cultural perspectives, and experiences shaped the selection, interpretation, and organization of concepts within the framework.

## 4 Rural IPV as an Ecology of Constraint

### 4.1 Societal: Rural Infrastructure and Geography

The vastness of rural geography and the lack of public transit infrastructure limit women's access to IPV‐crisis services. It is estimated that more than 25% of rural women live over 40 miles from the nearest IPV resource center, in comparison to less than 1% of urban women (Peek‐Asa et al. 2011). Moreover, rural IPV resources often serve multiple counties and offer fewer on‐site shelter services (Peek‐Asa et al. 2011), while operating under severe financial constraints (Eilbert and Luthern 2025; Luthern 2026). Survivors have reported that such services are frequently inaccessible due to transportation constraints and large distances from their home (Farhall et al. 2020; Wood et al. 2025). Accessibility is further compromised by severe weather, such as flooding, wildfires, or snowstorms, prompting road closures that prevent both survivors from reaching support services and advocates from delivering essential outreach and emergency care (National Academies of Sciences, Engineering, and Medicine 2024). Furthermore, some rural survivors may be unaware that IPV‐specific resources exist or may not know how to access them, reflecting limited visibility of available services and reduced outreach in geographically isolated communities (Robinson et al. 2021; Sabri et al. 2015). While IPV‐crisis services and domestic violence shelters strive to serve as beacons of safety, systemic underfunding and critical infrastructure gaps severely limit their capacity to meet the needs of rural women.

In addition to sparse advocacy services, rural healthcare infrastructure is facing unprecedented strain. Between 2005 and 2025, nearly 200 rural hospitals within the United States either closed entirely or were converted into outpatient facilities (Cecil G. Sheps Center for Health Services Research 2026). It is projected that these closures will continue, with more than 700 rural hospitals across the country at imminent risk of closure (Rural Hospitals At Risk Of Closing 2026), further restricting healthcare access and, importantly, opportunity for IPV disclosure and help‐seeking. Following incidents of violence, women may need to seek medical care for their injuries or assault, often in the emergency department (Davidov et al. 2015). Women experiencing IPV commonly present to emergency departments with injuries ranging from contusions, lacerations, and fractures to more severe injuries, including strangulation, TBI, and sexual assault. Head, neck, and facial injuries are common, and non‐fatal strangulation is linked with negative neurological outcomes, including hypoxic brain injuries, loss of consciousness, and seizures (National Academies of Sciences, Engineering, and Medicine 2024). For survivors in rural environments, medical care may now be many miles away, making it financially or logistically impossible to reach. Concurrently, this also increases response times for ambulance services, delaying access to critical on‐site emergency care for victims of physical violence (Jonk et al. 2023; Miller et al. 2020).

### 4.2 Community: Anonymity, Patriarchal Norms, and Racism

Within small towns that often make up rural communities, rural IPV survivors are restricted by lack of anonymity. In these close‐knit communities, social networks are deeply intertwined, and an abuser can maintain a well‐established public reputation, have personal relationships with law enforcement, or familial ties to prominent community figures (Campo and Tayton 2015; Farhall et al. 2020; Youngson et al. 2021). Women may also personally know individuals within the community, such as the medical receptionist or police officer (Eastman et al. 2007; Youngson et al. 2021). Such concerns about anonymity, community stigma, and town gossip may prevent women from seeking help from law enforcement or police, instead relying on informal sources (Ragusa 2017). Consequently, a survivor's attempt to seek help or report abuse is rarely confidential; instead, it risks immediate community exposure and severe retaliatory violence from the partner. This lack of privacy can be weaponized by the abuser, turning community spaces that should offer safety into areas of forced silence and compliance to the abuser.

Additionally, many rural communities are characterized by longstanding patriarchal gender norms that, while present across all geographic settings, may be reinforced by geographic isolation, close‐knit social networks, and cultural conservatism. These interacting factors can normalize male dominance, silence women, and compound other structural barriers to safety and help‐seeking (DeKeseredy and Rennison 2020; Silva 2022). Survivors have reported that these community norms minimized their partner's abuse and coercive control tactics, while simultaneously perpetuating the belief that a “good wife” stays silent (Crossman and Hardesty 2018; Towns and Adams 2016). This rural‐specific manifestation compounds other structural barriers. Patriarchal norms foster a dominant behavioral dynamic, not only between the woman and the abuser but also when it comes to police response. This translates into police victim‐blaming by insinuating the survivor did something to deserve the abuse and minimizing the violence as a private domestic matter to be resolved entirely within the home (Belisle et al. 2024; DeJong et al. 2008; Gezinski 2022). This response is inconsistent with the framework that governs institutional and community response to IPV as mandated in the U.S. Family Violence Prevention and Services Act of 1984 (Billings 2024).

For many survivors, these gendered expectations intersect with structural racism, historical trauma, economic marginalization, and other systems of oppression that further constrain opportunities for safety and help‐seeking (Luebke et al. 2021; Luebke et al. 2023). Consequently, survivors' decisions are shaped not only by gender norms but also by broader social, political, and historical contexts that shape institutional responses, community norms, and the availability and accessibility of formal and informal sources of support.

These intersecting systems may be particularly noticeable in rural communities characterized by racial segregation, limited culturally responsive services, or histories of structural inequity, where systemic racism and cultural biases may further compound minoritized survivors' well‐founded fears of hostile law enforcement encounters or discriminatory institutional responses (Brown et al. 2021; Luebke et al. 2023; Sharpless et al. 2024). These well‐founded concerns may act as a powerful deterrent to seeking help from law enforcement and other formal support systems.

Advocacy organizations are also affected by the rural environment. Rural‐based organizations balance confidentiality and community visibility. While an increased awareness of services is vital for women experiencing violence, it also increases the likelihood of compromised confidentiality for the survivor. In communities where anonymity is already compromised, advocacy organizations face additional hurdles to protecting their survivors, including keeping on‐site shelter safe and discreet, maintaining highly confidential relationships with hotels that provide emergency housing, and with advocates themselves, who are often members of the community they serve (Statz et al. 2022). Help‐seeking actions, such as parking outside an agency or meeting with a local advocate, can become public knowledge through small‐town social circles. This visibility exposes the survivor to social judgment and, more critically, to immediate retaliatory violence from an abuser monitoring her movements. Advocacy organizations also face significant funding and staffing hurdles, where they serve a large geographic area with limited federal and state funds, few donors, and a restricted local labor force from which to employ qualified advocates (Eastman and Bunch 2007; Peek‐Asa et al. 2011).

### 4.3 Relational: Coercive Control and Dependence

At the relational level, the immediate dynamic between a survivor and her partner is where her day‐to‐day reality lives. Coercive control is utilized by an abuser to limit a person's individual autonomy through repeated tactics of degradation, threats, surveillance, and restriction of resources, such as money, family, or work (Lohmann et al. 2024). The rural environment itself can amplify coercive control. Abusers can isolate the survivor through known lapses in confidentiality, invalidate the abuse by reinforcing their community reputation, or withhold transportation and access to neighboring areas (Campo and Tayton 2015; Crossman and Hardesty 2018; Farhall et al. 2020; Wood et al. 2025).

Women may also experience financial, logistical, and caregiving dependencies that make leaving an abusive relationship considerably more difficult. The poverty rate in rural areas is 15.4%, as compared to 11.9% in metropolitan areas (Farrigan 2026). Financial hardship can be exacerbated by limited employment opportunities in small towns, leaving residents unemployed or underemployed (Slack et al. 2020). This can foster a deep dependence to an abuser for financial stability and, subsequently, family basic needs including food and shelter. For relationships with children, women face immense pressure to ensure comfort and stability for their children, including providing for their basic needs. Survivors often cite children as being a complicating factor to leaving an abusive relationship, either acting as a facilitator or inhibitor (Rhodes et al. 2010; Sani and Pereira 2020). For mothers in rural environments, the decision to leave an abuser is particularly challenging if healthcare and advocacy services are many miles away from their child's school, home, and community. Additionally, they may also rely on their abuser financially and logistically. Children can also be used as part of coercive control, where abusers threaten to contact Child Protective Services or to take the children away (Rhodes et al. 2010). Ultimately, these relational dynamics work to entrap survivors, even when resources and disclosure opportunities exist.

### 4.4 Individual: Fear, Stigma, and Risk Perception

Individual‐level factors, including fear, perceived risk, and anticipated consequences, can substantially shape survivors' help‐seeking decisions. Disclosing violence, seeking formal support, or attempting to leave an abusive relationship can carry substantial risk. Separation and attempts to leave are well‐documented risk factors for intimate partner homicide, with evidence indicating that the period surrounding separation can be particularly dangerous for women experiencing IPV (J. C. Campbell et al. 2003). Survivors may therefore consider their perceived immediate risk of harm, including retaliatory or escalating violence, when calculating help‐seeking decisions.

Alongside perceptions of immediate risk, survivors may also weigh fear of continued violence, children's safety, financial insecurity, disruption of family relationships, and other consequences (Farhall et al. 2020; Heron et al. 2022; Sabri et al. 2022). Stigma and anticipated negative responses may further influence survivors' willingness to disclose IPV. Feelings of shame, guilt, embarrassment, self‐blame, and anticipatory judgment or disbelief from people to whom they disclose can act to silence survivors from sharing their abuse (Dipela and Ndhlovu 2025; Heron and Eisma 2021; Shorey et al. 2023). Together, fear, stigma, and perceptions of risk can influence how a survivor navigates IPV.

## 5 From Barriers to Constrained Agency

When structural infrastructure deficits, community cultural norms, relational control tactics, and internalized beliefs converge in the lives of women experiencing abuse, they do not merely act as passive barriers to care; rather, they actively produce a state of constrained agency, forcing rural survivors to make calculated compromises between accessing medical care, utilizing crisis resources, and preserving their immediate safety. Within the social sciences and gender‐based violence scholarship, constrained agency posits that an individual's independent actionable choices are limited or constrained by structural factors and social contexts (C. Campbell and Mannell 2016). In simple terms, constrained agency is a state of stripped independence where a survivor is forced to make decisions that directly conflict with her personal desires, beliefs, or long‐term safety, highlighting that these decisions are rarely simple.

Constrained agency is heavily documented throughout qualitative IPV literature, where women discuss themes of how they “can't leave” or “had to stay,” based on various aspects of their lives and surroundings (Crump 2020; McKinley and Liddell 2022; Wood et al. 2025). The absence of transit, lack of anonymity, and scarcity of resources leave survivors, particularly in rural settings, with no unpenalized options when making decisions regarding healthcare access, safety seeking, or disclosing violence. Autonomy and free choice are systematically restricted by the social ecology.

## 6 The Rural Ecological Framework of Intimate Partner Violence

The RE‐IPV is guided by the four ecological levels of the traditional SEM: societal, community, relational, and individual factors (Heise 1998). Rather than treating these levels as discrete, descriptive categories, RE‐IPV reconceptualizes them as dynamic, interacting processes that cumulatively restrict a woman's individual agency. The framework reconfigures these ecological levels as a downward funnel to visually and theoretically represent how interacting rural conditions progressively narrow the range of options available to survivors. Within this structure, constrained agency represents the process through which these converging ecological forces shape survivors' subsequent service navigation. The RE‐IPV model is presented in Figure 1.

**Figure 1:**
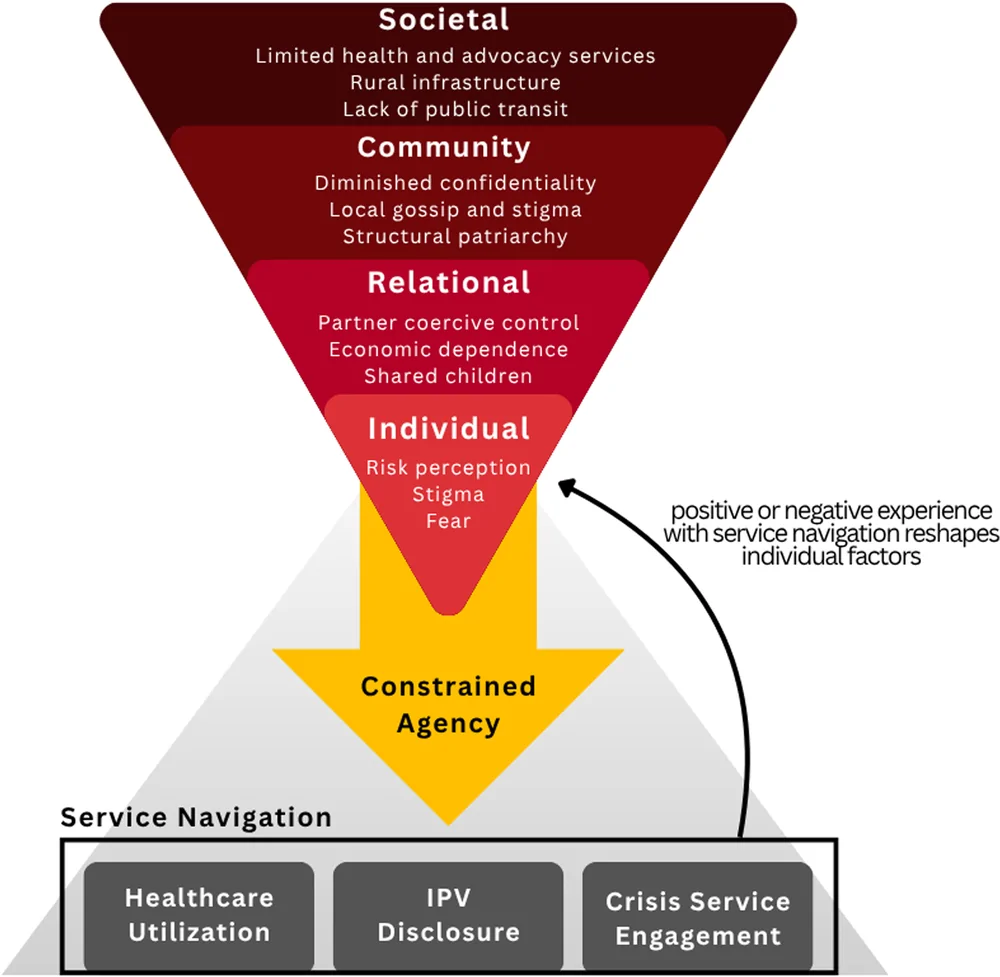
Rural ecological framework of intimate partner violence (RE‐IPV). The model illustrates how multi‐layered rural ecological factors converge to produce constrained agency, through which service navigation occurs within a dynamic feedback loop that continuously reshapes future help‐seeking decisions.

### 6.1 The Rural Ecology: Societal, Community, Relational, and Individual Factors

The societal tier establishes the structural parameters that shape all subsequent levels of the framework. It encompasses the overarching cultural, governmental, and structural matrix that dictates the options available to women and actively shapes the micro‐ecologies within it (Heise 1998). In the RE‐IPV model, this tier is operationalized through macro‐level disparities in rural infrastructure allocations and systemic inequities in advocacy service funding. Crucially, these societal conditions are not merely contextual; they actively determine whether help is geographically reachable, financially feasible, and institutionally available, often before a woman has even made a decision about seeking care.

The community tier represents the immediate social and physical environment in which a woman lives, the rural setting itself. This tier translates societal‐level structural conditions into concrete, everyday realities that women must navigate. In this layer, rural‐specific factors exert additional pressure onto the survivor; these include threats to confidentiality, community gossip, and a culture rooted in patriarchy (Brown et al. 2021; Campo and Tayton 2015; DeKeseredy and Rennison 2020; Towns and Adams 2016). These community‐level factors compound the pressures on women navigating violence, making help‐seeking not just difficult but actively dangerous.

The relational tier captures the immediate dynamic between the survivor and the person causing her harm. This is the space where societal and community constraints become intensely personal. Partner coercive control, economic dependence, and mutually shared children, all influenced by societal and community factors, work collectively to shape how the survivor perceives her options and ultimately how she responds (Clements et al. 2022; Crossman and Hardesty 2018; Wood et al. 2025). Together, the societal, community, and relational tiers converge on the individual level, which is the survivor herself.

The terminal point of the compounding ecological factors is the individual survivor. Crucially, the framework positions fear, stigma, and risk perception not as individual flaws or deficits, but as direct psychological consequences of the greater ecology, including patriarchal norms breeding stigma, coercive control fostering fear, and geographical isolation as well as logistical barriers shaping perceptions of safety. Along with societal, community, and relational factors, the individual tier is where all external pressures land and concentrate, shaping a survivor's capacity for autonomous decision‐making. Thus, the individual tier is not the source of the problem, but the site where the problem is more acutely felt.

### 6.2 Manufactured Constrained Agency

As these multi‐layered ecological factors converge with the individual tier, they actively manufacture a state of constrained agency for the survivor. Constrained agency is operationalized as individual decision‐making that is exercised within societal, community, relational, and individual limitations (C. Campbell and Mannell 2016). Fundamentally, constrained agency differs from learned helplessness. It is a structural condition produced by external forces rather than an individual deficit. When a rural survivor makes decisions that appear to conflict with her long‐term safety, such as remaining with an abuser, not seeking care for injuries, or not disclosing violence, the model reframes these actions as highly calculated compromises grounded in the realities of her surroundings. Ultimately, RE‐IPV positions constrained agency as arising from the rural ecology, and helps to explain how ecological conditions shape and restrict survivors' decision‐making and navigation of available services. Her decisions are not a reflection of individual pathology but of a landscape in which all available options may come at a substantial cost.

## 7 Service Navigation as a Recursive Process

As ecological conditions constrain the options available to them, rural survivors make complex decisions about whether, when, and how to navigate available services (Amel Barez et al. 2022; Spangaro, Herring, et al. 2016; Spangaro, Koziol‐McLain, et al. 2016; Wood et al. 2025). Within the RE‐IPV framework, service navigation is separated into three domains: healthcare utilization, seeking violence advocacy services, and whether to disclose their experience of violence to a professional. While existing frameworks often treat these endpoints as a linear conclusion to help‐seeking, the RE‐IPV model maps a critical secondary relationship; a direct feedback loop exists between institutional service encounters and the survivor's individual ecological tier. This feedback loop reflects the phenomenon of institutional betrayal, wherein systems designed to provide safety cause harm, reinforcing survivors' mistrust and reluctance to see future help (Lahav et al. 2026).

For a survivor, seeking advocacy services or disclosing violence represents a monumental, high‐risk decision. The experience when navigating these resources or disclosing their history of violence can either be positive or negative, thus fundamentally reframing an individual's fear and stigma, and ultimately dictating their future service navigation. This is particularly consequential when the experience is negative, such as racist or dismissive police responses (Belisle et al. 2024; Gezinski 2022), non‐empathetic or judgmental reactions from healthcare providers (HCPs) (Heron and Eisma 2021), or uninvolved advocacy responses. Survivors report that such responses make them less likely to seek help in future, as service navigation and disclosure now carry the added cost of compromising their emotional safety and amplifying perceived risk. Thus, the feedback loop captures a recursive process. Negative institutional encounters reinforce stigma and fear at the individual level, which in turn deepens constrained agency and shapes future help‐seeking decisions. Conversely, positive encounters lessen internalized fears and stigmas, increasing the likelihood for continued service navigation (Goodman et al. 2023).

## 8 Heterogeneity Within Rural Ecologies

Importantly, ecological factors do not operate uniformly across all survivors. Rather, structural barriers interact and reinforce one another across ecological levels, producing distinct experiences of constrained agency depending on survivors' social, cultural, historical, and political contexts. For women from racially and ethnically minoritized communities, including Black, Indigenous, Latina, and Somali women, rural ecological constraints frequently intersect with structural racism, historical trauma, and institutional inequities. Consequently, constrained agency is shaped not only by rural geography and patriarchal norms but also by longstanding systems of exclusion that influence safety, trust, and access to services (C. Campbell and Mannell 2016; Luebke et al. 2021, 2023). Minoritized women face disproportionately higher rates of both severe IPV and partner homicide, with the highest disparities concentrated among Indigenous and Black women (Petrosky et al. 2017; Zhang Kudon et al. 2026). These intersecting structural forces contribute to community‐level constraints, including anticipated discrimination, well‐founded distrust of institutions, historical trauma, and prior experiences of institutional harm, thereby narrowing the range of safe and acceptable help‐seeking options available to survivors (Luebke et al. 2021, 2023; Robinson et al. 2021). In historically White or deeply segregated rural communities, constrained agency may be further intensified by compounded social isolation, fewer culturally responsive services, and heightened visibility within close‐knit communities. This perspective builds on ecological and intersectional scholarship by emphasizing the importance of broader structural conditions in understanding survivors' experiences of IPV (Cardenas et al. 2024; Khan and Akram 2025).

Historical, political, and cultural contexts further shape how survivors experience the ecological factors described in the RE‐IPV framework. For Indigenous women living on or near Tribal Nations, help‐seeking may be further influenced by the enduring impacts of colonization, fragmented criminal jurisdiction, chronic underfunding of Tribal services, and the complex interactions among Tribal, state, and federal governments. These structural conditions can create inconsistent legal protections, fragmented service delivery, and uncertainty surrounding criminal justice responses despite the inherent sovereignty of Tribal Nations (Jock et al. 2022; Luebke et al. 2021). Similarly, Black and Indigenous survivors may experience well‐founded distrust of formal institutions because of historical and contemporary experiences of systemic racism, institutional betrayal, and discriminatory responses to violence (Luebke et al. 2023). Collectively, these examples demonstrate that ecological processes are not experienced uniformly across populations. Rather, structural inequities shape the contexts in which survivors navigate violence, evaluate available options, and seek support. Accordingly, application of the RE‐IPV framework should explicitly consider how colonization, fragmented jurisdiction, systemic racism, and other structural inequities influence survivors' ecological contexts and constrain help‐seeking.

Pregnant and postpartum women also have more complex ecologies in rural environments as their own individuality intersects with community‐ and structural‐level factors that influence their IPV experience and access to care. Pregnancy presents a unique time period in a woman's life where she is accessing the health system regularly for prenatal appointments, which provide an opportunity to screen and address IPV. It is recommended that women see a physician 6–10 times during the course of their pregnancy, with 1–2 visits postpartum (Peahl et al. 2025); however, rural maternity care services are also closing nationwide, creating “maternal care deserts.” Approximately 35% of US counties are classified as maternal care deserts, meaning they have no hospital or birth center offering essential prenatal and birth health services (Stoneburner et al. 2024). This affects more than 2.3 million women of childbearing age and 150,000 births annually (Stoneburner et al. 2024). For pregnant women also experiencing IPV, this places them in an impossible position where they may have to rely on their abuser for transportation or financial stability to make these appointments (Gurzenda et al. 2024). Living in rural communities with barriers to access healthcare means that pregnant women are geographically isolated from care. Geographical isolation is also worsened when women fear that disclosure may lead to their abuser and family knowing about their reporting (Riddell et al. 2009). To leave or report their abuser would leave them quite literally stranded. Ultimately, the erosion of rural maternity care not only exacerbates acute physical morbidity but systematically eliminates vital, confidential opportunities for IPV disclosure and professional intervention.

## 9 Implications for Practice and Policy

### 9.1 Nursing and Healthcare Practice Implications

The RE‐IPV framework offers a conceptual lens for understanding how rural ecological conditions shape survivors' interactions with healthcare systems. For nurses and other healthcare professionals, its central contribution is to shift attention from individual help‐seeking behaviors to the structural, community, and relational conditions within which those behaviors occur. Delayed disclosure, missed appointments, declining referrals, or remaining with an abusive partner may reflect constrained options rather than uncooperativeness, disengagement, or an unwillingness to seek assistance. Recognizing these conditions enables HCPs and legal systems to move beyond viewing survivor decisions in isolation, supporting more responsive, trauma‐informed, and survivor‐centered approaches to care that honor autonomy and individual needs (Tarzia et al. 2020).

Improving outcomes requires interventions addressing broader ecological influences alongside individual factors. At the practical level, routine IPV screening must incorporate private assessment environments and referrals accounting for transportation challenges, childcare obligations, and privacy concerns. Additionally, emerging technology‐based IPV interventions provide survivors with tailored safety planning and connections to resources to help bridge situational gaps. For example, *MyPlan*, a digital safety decision aid developed for IPV survivors in the United States, was associated with reduced decisional conflict and increased use of helpful safety behaviors (Glass et al. 2017). A subsequent randomized controlled trial of the *MyPlan* app among college women experiencing IPV found improvements in safety decision‐making and reductions in reproductive coercion (Glass et al. 2022). HCPs may consider incorporating *MyPlan* and similar safety‐planning tools into practice to aid in meeting the individual needs of rural survivors, though further empirical evidence is needed to evaluate their effectiveness in this context.

Multidisciplinary professionals must be trained in trauma‐informed care and cultural humility to support trusting, culturally safe healthcare environments, thus increasing subsequent IPV disclosure (Spangaro, Herring, et al. 2016; Spangaro, Koziol‐McLain, et al. 2016). Importantly, the RE‐IPV framework's feedback loop highlights that healthcare encounters can shape survivors' willingness and perceived ability to seek assistance in the future. Supportive, nonjudgmental responses may foster trust and facilitate continued engagement, whereas dismissive or stigmatizing encounters may reinforce existing barriers. Advocacy services should demonstrate flexible delivery approaches, such as mobile applications, healthcare collaborations, and outreach clinics, to minimize geographic obstacles.

### 9.2 Policy and Structural Implications

Regarding policy, decision‐makers must prioritize expanding public transit, emergency transportation, and transit vouchers to address primary structural barriers. Sustainable funding is needed for rural healthcare facilities, domestic violence shelters, advocacy initiatives, and emergency transport services to reduce rural–urban discrepancies. Policies must also address rural poverty, housing insecurity, and financial dependence while developing strategies like independent reporting instruments and enhanced privacy protocols to protect confidentiality and promote survivor disclosure.

## 10 Conceptual Boundaries and Future Development

As a conceptual framework, RE‐IPV offers a way to understand how interacting rural ecological conditions shape survivors' agency and service navigation, while also identifying areas requiring further empirical examination and conceptual development. The RE‐IPV framework was developed through conceptual synthesis rather than primary empirical testing; therefore, the associations between the SEM levels and constrained agency require empirical validation in rural populations and settings. Moreover, rural communities are heterogeneous, and the experiences of survivors of IPV may differ according to the geographic location, culture, healthcare infrastructure, and the availability of community resources. Although RE‐IPV was developed within the US context, the framework may offer a conceptual foundation for examining similar rural ecologies and survivor resource navigation in other countries, including Australia and Canada. However, its applicability across contexts should not be assumed; further research is needed to examine how country‐specific policies, service infrastructures, cultural norms, and Indigenous histories and experiences shape their rural ecology and subsequent service navigation.

While the RE‐IPV framework highlights how structural and ecological conditions constrain survivors' decision‐making, it does not fully account for protective factors, personal resilience, or community strengths that support survivors and service navigation. Future research should examine the framework's applicability across diverse rural contexts and explore how it might be refined to account for conditions that both constrain and expand survivors' options, with implications for healthcare access, service delivery, and safety.

## 11 Conclusion

IPV remains a prominent, yet underrecognized, public health crisis. Women in rural settings face significant barriers to help‐seeking and service navigation that are shaped and often intensified by rural social and structural conditions. The RE‐IPV framework advances IPV scholarship by (1) using constrained agency to explain how interacting ecological conditions shape survivors' decisions and service navigation, (2) capturing the dynamic, recursive nature of help‐seeking, and (3) providing a rural‐specific lens that addresses the distinct structural realities of rural survivors' experiences. The framework offers a roadmap for researchers, clinicians, and policymakers seeking to understand and address the compounded barriers facing rural IPV survivors.

## Funding

The authors have nothing to report.

## Ethics Statement

Ethics approval was not required for this manuscript, as it presents a conceptual framework derived from literature synthesis without primary human subjects research.

## Conflicts of Interest

The authors declare no conflicts of interest.

## Artificial Intelligence Disclosure

Generative AI (Gemini) was used minimally for select sentence‐level grammar and language refinement. No AI tools were used for conceptual development, literature review, synthesis, original text generation, or figure creation. The authors assume full responsibility for the contents of the manuscript.

## Permission to Reproduce Material

The figure presented in this manuscript is original work created by the authors and does not require permission to reproduce material from other sources.

## Data Availability

Data sharing is not applicable to this article as no datasets were generated or analyzed during the current study.
